# Supplementary material for: Association of Vascular Endothelial Growth Factors (VEGFs) with Recurrent Miscarriage: A Systematic Review of the Literature
Source: Int J Mol Sci. 2023 May 29;24(11):9449. doi: 10.3390/ijms24119449 (PMC10253948; doi:10.3390/ijms24119449)
Supplement: Supplementary file 1 [file ijms-24-09449-s001.zip › ijms-2304564-supplementary.pdf]

SUPPLEMENTARY MATERIALS

Appendix S1 – Examples of Search Strategies

Embase (Ovid): 2000 to current

Date searched: 18.09.2021

1 exp Vascular endotherlial growth factor or VEGF or PIGF, or VEGF-B or VEGF-C or VEGF-D or VEGFR-1 or FLT-1 or VEGFR-2 or KDR or VEGFR-3 or FLT-4/133130

2 recurrent miscarriage* or recurrent pregnancy loss or spontaneous abortion.tw/54759

3 1 and 2/293

4 limit 3 to (english language and yr = 2000 – current)/282

MEDLINE

Date searched: 18.09.2021

1 exp Vascular endotherlial growth factor or VEGF or PIGF, or VEGF-B or VEGF-C or VEGF-D or VEGFR-1 or FLT-1 or VEGFR-2 or KDR or VEGFR-3 or FLT-4/102783

2 recurrent miscarriage* or recurrent pregnancy loss or spontaneous abortion.tw/11821

3 1 and 2/126

4 limit 3 to (english language and yr = 2000 – current)/119

Appendix S2 – Excluded Studies

Table S1- Reasons for the exclusions of studies

Author/Year	Study Title	Reason for exclusion
**Chai et al., 2013**	Expression of ATF4 and VEGF in chorionic villus tissue in early spontaneous abortion	The authors recruited cases of women with a history of spontaneous miscarriage. No reference was made about a personal history of recurrent miscarriages
**Choi et al., 2003**	Expression of Angiogenesis- and Apoptosis-Related Genes in Chorionic Villi Derived From Recurrent Pregnancy Loss Patients	The authors utilised semi-quantitative RT-PCR analysis and Norther Blot analysis to determine VEGF concentration and levels in cases and control. The authors did not utilise immunohistochemistry to in their methodology
**Dev et al., 2020**	Assessment of Maternal Serum Levels of Vascular Endothelial Growth Factor and Placental Growth Factor in Threatened Abortion: A Case Control Study	The authors defined cases as pregnant women with a threatened miscarriage. No reference was made about a personal history of recurrent miscarriages
**Keskin et al., 2015**	The relationship between the VEGF/sVEGFR-1 ratio and threatened abortion	The authors defined cases as pregnant women with a threatened miscarriage. No reference was made about a personal history of recurrent miscarriages
**Kutluer et al., 2012**	Low VEGF expression in conceptus material and maternal serum AFP and β-hCG levels as indicators of defective angiogenesis in first-trimester miscarriages	The authors defined cases as women experiencing incomplete miscarriages or intrauterine death. No reference was made about a personal history of recurrent miscarriages
**Pang et al., 2011**	Vascular Endothelial Growth Factor (VEGF) and the VEGF Soluble Receptor-1 (sFlt-1) in Chorionic Villus Tissue from Chinese Women with Early Recurrent Spontaneous Abortion	The authors utilised semi-quantitative RT-PCR analysis to determine VEGF concentration and levels in cases and control. The authors did not utilise immunohistochemistry to in their methodology
**Ugurlu et al., 2008**	The value of vascular endothelial growth factor, pregnancy-associated plasma protein-A, and progesterone for early differentiation of ectopic pregnancies, normal intrauterine pregnancies, and spontaneous miscarriages	The authors recruited cases of women with a history of spontaneous miscarriage or suspected ectopic pregnancy. No reference was made about a personal history of recurrent miscarriages
**Wyatt et al., 2021**	Evaluating Markers of Immune Tolerance and Angiogenesis in Maternal Blood for an Association with Risk of Pregnancy Loss	The authors recruited cases of women with a history of spontaneous miscarriage. No reference was made about a personal history of recurrent miscarriages

Appendix S3 – Bias Analyses of Included Studies

Table S2- Risk of bias: review authors' judgements about each domain for each included study. Scores arise from the Joanna Briggs Institute -Prevalence Critical Appraisal Checklist

Paper	*Q1*	*Q2*	*Q3*	*Q4*	*Q5*	*Q6*	*Q7*	*Q8*	*Q9*	*Q10*
Almawi et al., 2013	Y	Y	Y	Y	Y	Y	Y	Y	N/A	Y
Atalay et al., 2016	Y	Y	Y	Y	Y	Y	Y	Y	N/A	Y
Amirchaghmaghi et al., 2014	Y	N	Y	N	Y	Y	Y	Y	N/A	Y
Bagheri et al., 2017	Y	Y	Y	Y	Y	Y	Y	Y	N/A	Y
Banerjee et al., 2013	Y	Y	Y	Y	Y	Y	Y	Y	N/A	Y
**Gupta et al., 2019**	Y	Y	Y	Y	Y	Y	Y	Y	N/A	Y
He et al., 2016	Y	Y	Y	Y	Y	N	N	Y	N/A	Y
Lash et al., 2016	N	Y	N	N	Y	Y	N	Y	N/A	Y
Pang et al., 2013	Y	Y	Y	N	Y	N	N	Y	N/A	Y
Papamitsou et al., 2021	N	Y	Y	Y	Y	Y	Y	Y	N/A	Y
Sadekova et al., 2015	Y	Y	Y	N	Y	Y	Y	Y	N/A	Y
Scarpellini et al., 2019	Y	N	Y	Y	Y	Y	Y	Y	N/A	Y
Vuorela et al., 2000	Y	N	Y	Y	Y	Y	Y	Y	N/A	N

Abbreviations: Yes (Y), No (N), Unclear (UC)
1: Were the groups comparable other than the presence of disease in cases or the absence of disease in controls?
2: Were cases and controls matched appropriately?
3: Were the same criteria used for identification of cases and controls?
4: Was exposure measured in a standard, valid and reliable way?
5: Was exposure measured in the same way for cases and controls?
6: Were confounding factors identified?
7: Were strategies to deal with confounding factors stated?
8: Were outcomes assessed in a standard, valid and reliable way for cases and controls?
9: Was the exposure period of interest long enough to be meaningful?
10: Was appropriate statistical analysis used?

Figure S1- Summary of the risk of bias of included cohorts represented as an overall proportion, per question of the JBI tool

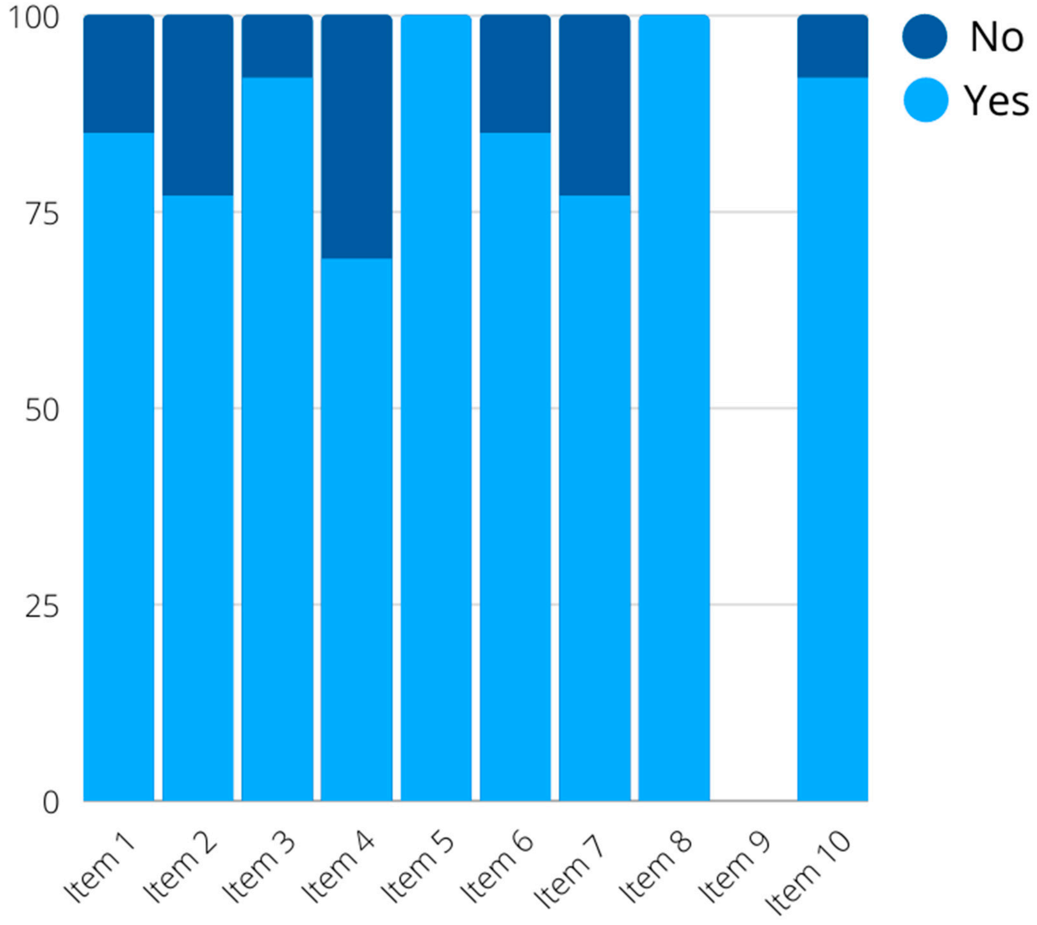

1: Were the groups comparable other than the presence of disease in cases or the absence of disease in controls?
2: Were cases and controls matched appropriately?
3: Were the same criteria used for identification of cases and controls?
4: Was exposure measured in a standard, valid and reliable way?
5: Was exposure measured in the same way for cases and controls?
6: Were confounding factors identified?
7: Were strategies to deal with confounding factors stated?
8: Were outcomes assessed in a standard, valid and reliable way for cases and controls?
9: Was the exposure period of interest long enough to be meaningful?
10: Was appropriate statistical analysis used?
